# The Proneural Molecular Signature Is Enriched in Oligodendrogliomas and Predicts Improved Survival among Diffuse Gliomas

**DOI:** 10.1371/journal.pone.0012548

**Published:** 2010-09-03

**Authors:** Lee A. D. Cooper, David A. Gutman, Qi Long, Brent A. Johnson, Sharath R. Cholleti, Tahsin Kurc, Joel H. Saltz, Daniel J. Brat, Carlos S. Moreno

**Affiliations:** 1 Center for Comprehensive Informatics, Emory University, Atlanta, Georgia, United States of America; 2 Pathology and Laboratory Medicine, Emory University School of Medicine, Atlanta, Georgia, United States of America; 3 Emory Winship Cancer Institute, Atlanta, Georgia, United States of America; 4 Department of Biostatistics and Bioinformatics, Emory University, Atlanta, Georgia, United States of America; The University of Chicago, United States of America

## Abstract

The Cancer Genome Atlas Project (TCGA) has produced an extensive collection of ‘-omic’ data on glioblastoma (GBM), resulting in several key insights on expression signatures. Despite the richness of TCGA GBM data, the absence of lower grade gliomas in this data set prevents analysis genes related to progression and the uncovering of predictive signatures. A complementary dataset exists in the form of the NCI Repository for Molecular Brain Neoplasia Data (Rembrandt), which contains molecular and clinical data for diffuse gliomas across the full spectrum of histologic class and grade. Here we present an investigation of the significance of the TCGA consortium's expression classification when applied to Rembrandt gliomas. We demonstrate that the proneural signature predicts improved clinical outcome among 176 Rembrandt gliomas that includes all histologies and grades, including GBMs (log rank test p = 1.16e-6), but also among 75 grade II and grade III samples (p = 2.65e-4). This gene expression signature was enriched in tumors with oligodendroglioma histology and also predicted improved survival in this tumor type (n = 43, p = 1.25e-4). Thus, expression signatures identified in the TCGA analysis of GBMs also have intrinsic prognostic value for lower grade oligodendrogliomas, and likely represent important differences in tumor biology with implications for treatment and therapy. Integrated DNA and RNA analysis of low-grade and high-grade proneural gliomas identified increased expression and gene amplification of several genes including GLIS3, TGFB2, TNC, AURKA, and VEGFA in proneural GBMs, with corresponding loss of DLL3 and HEY2. Pathway analysis highlights the importance of the Notch and Hedgehog pathways in the proneural subtype. This demonstrates that the expression signatures identified in the TCGA analysis of GBMs also have intrinsic prognostic value for low-grade oligodendrogliomas, and likely represent important differences in tumor biology with implications for treatment and therapy.

## Introduction

Glioblastoma (GBM) is the most common primary brain tumor, with 8700 new cases per year in the United States [1]. It is also the highest grade astrocytoma (WHO grade IV), with a truly dismal prognosis [2]. Following surgical resection, radiation therapy and temozolamide chemotherapy–the current therapeutic gold standard–mean survival is 60 weeks [3]. Perhaps more telling of its aggressive clinical behavior, survival of patients treated by surgical resection alone averages 13 weeks [4]. Lower grade gliomas (i.e. WHO grade II and III) account for an additional 2500 cases/year and are also ultimately fatal, but have slower growth rates and longer survival times (3–8 years). With rare exception, patients with lower grade tumors will eventually die due to progression to GBM.

The analysis of gene expression patterns in GBMs suggests that this histologic category may include distinct subtypes. Several groups have developed approaches for subtyping GBMs by gene expression signatures [5], [6]. *Verhaak et al* [6] used data from The Cancer Genome Atlas (TCGA) to describe four distinct subtypes of GBM (Proneural, Neural, Classic, and Mesenchymal) that are defined by gene expression signatures following unsupervised hierarchical clustering. TCGA gene expression subtypes have moderate similarity to those described by *Phillips et al* [7], who defined three transcriptional subtypes (proneural, proliferative, and mesenchymal). Although the TCGA analysis did not reveal differences in survival between subtypes, this may reflect the overall short survival period for this highly malignant neoplasm [8]. The TCGA data at this point does not contain any lower-grade gliomas. To assess whether the gene expression patterns uncovered by TCGA data could be used to segregate or predict survival of lower-grade gliomas, we applied these gene signatures to the NCI Repository for Molecular Brain Neoplasia Data (Rembrandt) dataset, which includes infiltrative gliomas with diverse histologies and includes grades II, III, and IV. The Rembrandt dataset used here contains 176 samples with long-term survival data, including 32 astrocytomas (WHO grades II and III), and 43 oligodendrogliomas (WHO grades II and III). Here, we demonstrate that the Proneural gene expression signature as defined by TCGA is enriched in gliomas with oligodendrogliomal differentiation. This signature also predicts improved outcome for oligodendrogliomas. Moreover, we have compared the expression patterns and DNA copy number of low-grade and high-grade proneural gliomas and identified genes and pathways associated with progression of this disease. Genes increased in proneural GBMs (PN-GBM) at both the DNA and RNA level included GLIS3, TGFB2, TNC, AURKA, and VEGFA. Network analysis of these changes in gene expression identified several regulatory hubs, including GLI1, RUNX2, MYC, BMP2, and NOTCH1.

## Materials and Methods

### Ethics Statement

All human subjects data was publicly available de-identified data from The Cancer Genome Atlas project and the Rembrandt database, and thus, not designated as human subjects research. No Institutional Review Board approval was required.

### Patient Datasets

Microarray and clinical data were acquired in un-normalized form from the Rembrandt [9] public data repository (https://caintegrator.nci.nih.gov/rembrandt/) using data available on November 11, 2009. Clinical data were derived from contributing center institutional diagnoses at Henry Ford Hospital, UCSF, Lee Moffitt Cancer Center, Dana Farber Cancer Center, University of Wisconsin, and NCI and are available as Table S1. Affymetrix HGU133 Plus 2.0 CEL files were normalized using the robust multi-array average (RMA) method [10] with default parameters from the Matlab Bioinformatics Toolbox. Of the 296 samples downloaded, 176 had associated survival data, 75 were oligodendrogliomas or astrocytomas with grades II or III, and 101 were GBMs. Copy number data was derived using Affymetrix 100K SNP arrays available from the Rembrandt public data repository. Of the 176 samples with survival data, 147 have associated SNP arrays for tumor tissue (85 GBMs, 34 Oligodendrogliomas grades II and III, and 28 Astrocytomas grades II and III).

### Data Analysis

To classify Rembrandt samples within the TCGA classification schema, Rembrandt data for the probes from the Affymetrix U133 Plus 2.0 GeneChip were mapped to the TCGA class signature genes using HUGO gene symbol and Entrez gene ID number. This comparison yielded an intersection of 1486 Affymetrix probe sets. Classification of the Rembrandt samples was then performed using prediction analysis for microarrays using the signature gene class centroids [11].

Comparisons of survival between different classes were performed using the log-rank test [12] as implemented in GenePattern 3.0 [13]. Survival was compared between each expression subtype and all others for all 176 samples, all grade II and III gliomas, and the subsets of the 32 astrocytomas, and 43 oligodendrogliomas. Unsupervised hierarchical clustering was performed using uncentered correlation and average linkage with gene and array centering and normalization using cluster [14] and javatreeview [15].

Differences in gene expression between subtypes were determined using the comparative selection marker module of GenePattern 3.0. Cutoffs for statistical significance were a Benjamini- Hochberg corrected False Discovery Rate (FDR) <0.05 and a minimum fold change >1.8. Statistical significance of Gene Ontology overrepresentation were determined by hypergeometric distribution using the DAVID database [16] and Ingenuity Pathway Analysis [17]. Gene Set Enrichment Analysis used the GSEA software [18] with an FDR cutoff of 0.25. Classes were analyzed using the curated c2.biocarta.v2.5.symbols and c2.kegg.v2.5.symbols databases and the gene ontology c5.all.v2.5.symbols and c5.bp.v2.5.symbols databases from the Molecular Signatures Database (MSigDB) at the Broad Institute [18]. Literature Lab software [19] version 2.9 (Acumenta, Inc., Boston, MA) was used to identify statistically significant associations of gene lists with Pathways and MESH terms, and to compare gene lists against one another.

Cox proportional hazards (PH) models were used to examine the association between patient survival and four subtypes after adjusting for patient age at diagnosis as well as 1p/19q status whenever applicable. We note that 1p/19q deletion is present only in Oligodendrogliomas; hence, 1p/19q deletion status was not adjusted for in cases with Astrocytoma or GBM.

## Results

Unsupervised hierarchical cluster analysis of gene expression data derived from TCGA GBM samples resulted in four distinct gene expression subtypes: Neural, Proneural, Classical, and Mesenchymal. This classification utilized an integrated analysis of three gene expression platforms to identify a set of genes that is consistently and variably expressed among the TCGA samples. Expression measurements from the Affymetrix HT-HG-U133A, the Affymetrix Human Exon 1.0 ST, and a custom Agilent array, were combined by mapping to a transcript database 11,861 total genes. Of these, 1740 were reliably expressed across platforms, with 840 identified as class signature genes by ClaNC analysis [20]. The full details of the TCGA cluster analysis are available elsewhere [8].

**Figure 1:**
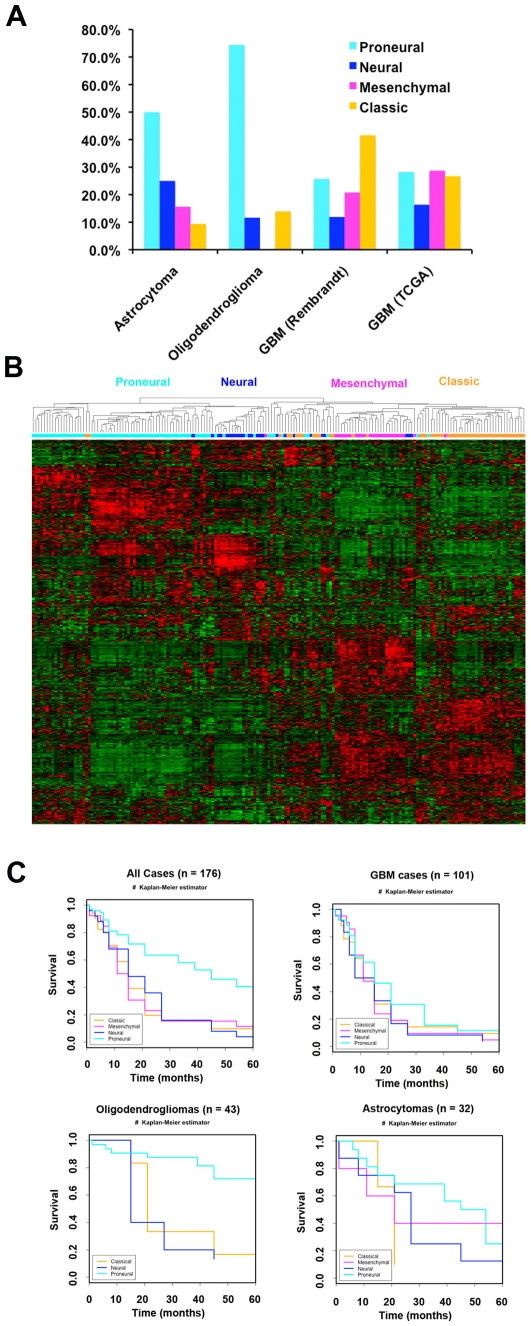
The proneural subtype is enriched in oligodendrogliomas and has longer survival. (**A**) Unsupervised hierarchical clustering of 176 Rembrandt samples using TCGA classification genes identifies the four major subtypes: Proneural (cyan), Neural (blue), Mesenchymal (magenta), and Classic (orange). (**B**) Kaplan-Meier survival samples shows the Proneural subtype has significantly better survival for all cases (n = 176, p = 1.16e-6), and oligodendrogliomas (n = 43, p = 1.26e-4), but not for astrocytomas (n = 32, p = 0.3), or GBM cases (n = 101, p = 0.588).

Total gene expression and clinical survival data were downloaded from the Rembrandt website (https://caintegrator.nci.nih.gov/rembrandt/). To map the 176 Rembrandt samples with survival data to the relevant TCGA molecular subtypes, we extracted the gene symbols from the 840 gene centroid developed previously [6]. Gene symbols and corresponding entrez gene ID numbers were used to filter the Rembrandt Affymetrix U133Plus2.0 GeneChip data, resulting in a total of 1486 probes corresponding to 819 genes. These data were analyzed by supervised hierarchical clustering (Figure 1) and visual inspection indicated that the four subtypes are readily discernible in the Rembrandt dataset. A composite dataset corresponding to these 819 genes was then assembled including both the TCGA and Rembrandt datasets. A shrunken centroid model was trained on the TCGA data using PAM software [11] and tested by leave-one-out-cross-validation (Table 1, 148/160 correct = 92.5% classification accuracy). We next applied the centroid model to the Rembrandt dataset to classify each of the samples to one of the four molecular subtypes (Table 2 and Figure 1A). Classification of GBM samples in the Rembrandt dataset mirrored those previously observed in the TCGA analysis. We observed that the proneural class dominated the oligodendrogliomas grade II and III samples (32/43 = 74%), and that the mesenchymal class was completely absent in the oligodendrogliomas (Figure 1A). Although the proneural class was also enriched somewhat in astrocytomas, it was less dramatic, and all gene expression subtypes, including mesenchymal, were represented. The classic signature was more common in GBM samples than in grade II and III samples. Classifications, class probabilities, pathology, and survival data for all 176 samples included in this analysis are provided in Table S2. The complete set of clinical data for all of the Rembrandt samples used in this analysis downloaded in November, 2009 is given in Table S1. Unsupervised hierarchical clustering of these 176 samples using these 819 genes shows that they cluster into the four representative subtypes identified in the TCGA GBM dataset (Figure 1B).

**Table 1:** Classification accuracy of subtype predictor on the training set of TCGA samples generated using Prediction Analysis of Microarrays (PAM) software.

*CV Confusion Matrix (Threshold = 2.66891)*					
True\Predicted	Classic	Mesenchymal	Neural	Proneural	Class Error rate
Classic	43	4	0	0	0.085
Mesenchymal	2	40	1	0	0.070
Neural	0	1	28	1	0.067
Proneural	1	0	2	37	0.075

Overall prediction accuracy was 148/160 = 92.5% correct.

**Table 2:** Classification of Rembrandt samples using the TCGA molecular subtype gene signatures derived from the PAM classifier.

*Subtype*	*GBM*	*Oligodendroglioma*	*Astrocytoma*	*All*
Classic	42	6	3	51
Mesenchymal	21	0	5	26
Neural	12	5	8	25
Proneural	26	32	16	74
**Total**	101	43	32	176

The Proneural subtype dominates oligodendriogliomas and there are no mesenchymal subtype in the oligodenroglioma samples.

To examine if there were any differences in survival for the entire set of 176 classified Rembrandt samples, we performed a Kaplan-Meier analysis (Figure 1C). The 74 samples in the Proneural class had significantly better outcome by the log rank test (p = 1.16e-6). The other three gene expression classes did not show appreciable survival differences. We next examined whether gene expression class was predictive of outcome in grade II and grade III gliomas, including both oligodendrogliomas and astrocytomas in the analysis. We found that the proneural subtype predicted significantly better outcome in lower grade gliomas (p = 0.000265, Figure S1). To determine whether the prognostic value of the Proneural subtype was independent of histology and grade (GBM, oligodendroglioma, or astrocytoma), we performed a similar analysis on each of these subsets. The Proneural subtype demonstrated improved outcome for oligodendrogliomas (grades II and III, p = 1.26e-4), but not astrocytomas (grades II and III, p = 0.3) or GBM (grade IV, p = 0.588). *Noushmehr et al* [21] has recently identified a specific subtype of diffuse glioma based on its CpG methylation pattern that is enriched in the proneural subtype, in oligodendrogliomas, and in all low-grade gliomas, which has significantly improved outcome, corroborating our findings.

We next performed a multivariate Cox Proportional Hazard analysis of expression subtypes, adjusting for age and 1p/19q deletion status (**Table 3**). Only 152 samples could be included in this analysis, as 24 samples lacked SNP/copy number data for assessing 1p/19q status. As expected, the classic (HR = 6.25; p = 0.0100) and neural (HR = 23.28; p = 0.0001) subtypes of oligodendrogliomas had significantly worse outcome compared to the proneural subtype. For astrocytomas, the classic subtype had significantly worse outcome than proneural cases (HR = 8.48; p = 0.0059), but the mesenchymal and neural subtypes did not exhibit significantly different outcome from proneural samples.

**Table 3:** Cox proportional hazards (PH) models were used to examine the association between patient survival and four subtypes after adjusting for patient age at diagnosis as well as 1p/19q status whenever applicable.

	*All Cases*	*GBM*	*Astrocytomas*	*Oligodendrogliomas*
*Number of cases*	152	101	32	36
*Subtype*				
*Classic*	1.96 (CI: 1.23, 3.13); **p = 0.0045**	1.17 (CI: 0.69, 1.96); p = 0.563	8.48 (CI: 1.85, 38.85); **p = 0.0059**	6.25 (CI: 1.55, 25.19); **p = 0.0100**
*Mesenchymal*	2.68 (CI: 1.51, 4.76); **p = 0.0008**	1.56 (CI: 0.85, 2.85); p = 0.150	1.04 (CI: 0.29, 3.76); p = 0.9537	N/A
*Neural*	2.38 (CI: 1.38, 4.11); **p = 0.0018**	1.57 (CI: 0.78, 3.17); p = 0.207	1.91 (CI: 0.72, 5.07); p = 0.1963	23.28 (CI: 4.63, 116.98); **p = 0.0001**
*Age (≥5 vs <55)*	3.14 (CI: 2.12, 4.64); **p<0.0001**	2.90 (CI: 1.84, 4.55); **p<0.0001**	6.79 (CI: 2.77, 16.66); **p<0.0001**	4.97 (CI: 1.58, 15.70); **p = 0.0062**
*1p/19q (deletion vs. no deletion)*	0.42 (CI: 0.16, 1.05); p = 0.0634	N/A	N/A	2.23 (CI: 0.59, 8.46); p = 0.2380

Shown are Hazard Ratios (HRs) plus 95% confidence intervals (CI) and associated p-values. Significant p-values (<0.05) are shown in bold font. For the Cox PH models, Proneural was used as the reference group for the subtype variable, patient age was dichotomized as <55 or ≥55 with <55 as the reference group, and no deletion in 1p/19q status was used as the reference group. Cox PH multivariate analysis was performed for all cases, cases with Astrocytoma, cases with GBM and cases with Oligodendrogliomas, respectively. We note that 1p/19q deletion is present only in Oligodendrogliomas; hence, 1p/19q deletion status was not adjusted for in cases with Astrocytoma or GBM.

To make sure that none of the classic or neural oligodendrogliomas were misclassified small cell GBMs, we carefully examined patient age, chromosome 10 loss, and EGFR amplification status. Of the six classic and five neural oligodenrogliomas, none of the neural and only two of the classic oligodendrogliomas had high patient age (>70), chr10 loss, and/or EGFR amplification. We reanalyzed the survival data excluding these two cases (HF1150 and HF0510), and none of the statistical findings were affected, except that the p-value for HR comparing classic and proneural oligodendrogliomas increased to 0.0595 (marginally significant) from 0.0100 (significant), which is not too surprising given that the sample size of the oligodendrogliomas was reduced by two.

### Gene Expression Analysis

**Figure 2:**
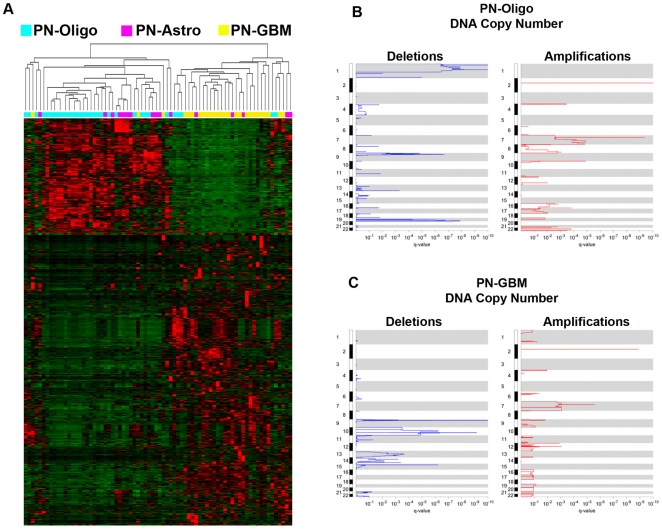
RNA and DNA copy number analysis of low- and high-grade proneural gliomas. (**A**) Supervised hierarchical clustering of Proneural GBM, Proneural Oligodendrogliomas, and Proneural Astrocytomas using 847 probes significantly different between these subtypes based on differential marker analysis (FDR <5%, 1.8 Fold change). (**B**) Amplification and Deletion GISTIC Analysis of Proneural GBM and Proneural Oligodendrogliomas. Affymetrix SNP 6.0 data from the Rembrandt dataset was analyzed for copy number gain and loss using the GISTIC algorithm in GenePattern 3.0. A total of 26 Proneural GBM and 32 Proneural Oligodendroglioma samples were included in the analysis. No significant copy number changes were identified in the set of 14 Proneural Astrocytomas.

To investigate mechanisms underlying the difference in survival associated with the Proneural subtype, we performed several differential gene expression analyses. First we compared the set of proneural lower grade gliomas (both oligodendrogliomas and astrocytomas; PN-OA) to proneural GBMs (PN-GBM). In addition, because of the markedly improved survival of proneural oligodendrogliomas (PN-Oligo), we compared the gene expression of PN- Oligo to PN-GBM, proneural astrocytomas (PN-Astro), neural oligodendrogliomas (N-Oligo), and classic oligodendrogliomas (C-Oligo). We also compared proneural oligodendrogliomas to the combined set of classic and neural oligodendrogliomas. Using minimum fold-change cutoff of 1.8 and a Benjamini- Hochberg corrected False Discovery Rate (FDR) <0.05, we identified 779 probes differentially regulated between PN-Oligo and PN-GBM (Table S3), and 576 probes differentially regulated between PN-OA and PN-GBM (Table S4). Venn diagram analysis of these two gene sets determined that 508 probes were in common, 68 were unique to the PN-OA vs PN-GBM comparison, and 271 were unique to the PN-Oligo vs PN-GBM comparison. Thus, the union of these comparisons resulted in 847 differentially expressed probes. A hierarchical clustering of the PN-Oligo, PN-Astro, and PN-GBM samples using these 847 differentially expressed probes is shown in **Figure 2A**. With identical false discovery and fold-change criteria, we found no significant probes differentially expressed between PN-Oligo and PN-Astro subtypes. However, we did observe 1508 probes differentially expressed between PN-Oligo and N-Oligo (Table S5), 2118 probes differentially expressed between PN-Oligo and C-Oligo (Table S6), and 190 probes differentially expressed between PN-Oligo and the combined set of N-Oligo and C-Oligo (Table S7). Complete results for these comparisons providing data on all probes is also available in Tables S3, S4, S5, S6.

### Copy Number Analysis

DNA Copy number data for the Rembrandt samples was calculated using GenePattern version 3.2.0. Segmentation of raw copy numbers was calculated using the GLAD module (version 2). Significance analysis of amplifications and deletions was performed using the GISTIC method [22] (version 3), with amplification/deletion threshold 0.1 and minimum segment size of four markers.

We identified a number of regions with significant amplifications or deletions in the PN-GBM and PN-Oligo subtypes (**Figure 2B**). It is worth noting that the PN-Oligo samples had highly significant frequency of co-deletion of chromosomes 1p36 and 19q13. While there were relatively few neural (n = 3) and classic (n = 5) oligodendrogliomas with SNP data, and no mesenchymal oligodendrogliomas, we did note that they generally contained either 1p deletion or 19q deletion, but not both deletions in any given tumor sample.

### Integrated Copy Number and Gene Expression Analysis

To gain insight into the progression of proneural samples, we performed an integrated analysis of DNA copy number and mRNA gene expression within the proneural subtype of the Rembrandt samples, specifically examining those changes between PN-OA and PN-GBM samples. We observed 66 probe sets corresponding to 52 genes that showed significant amplification or deletion, as well as significant differences in gene expression between these subtypes. A comprehensive list of the mRNA probes that intersect with GISTIC copy number analysis is given in Table S8, and a summary of the loci with both expression and copy number alterations between PN-GBM and PN-OA samples is provided in **Table 4**. Among these genes, of particular interest, were the Hedgehog pathway transcription factor GLIS3 (amplified at 9p23 and increased in PN-GBM), Meningioma 1 (MN1), RAS-like 10A (RASL10A), Src homology 2 containing transforming protein D (SHD), and Dishevelled associated activator of morphogenesis 2 (DAAM2), all amplified in PN-OA samples and increased in PN-OA relative to PN-GBM. Genes deleted in PN-OA samples and downregulated relative to PN-GBM included Epithelial membrane protein 3 (EMP3), Secreted phosphoprotein 1/Osteopontin (SPP1), and integrin-binding sialoprotein (IBSP). Also of note was the fact that multiple genes associated with the complement cascade (C1S, C1R, C1RL, F13A1, and CFI) were within the set of genes showing both copy number and expression changes between PN-OA and PN-GBM samples. All of these complement cascade genes showed higher expression in PN-GBM samples relative to PN-OA samples.

**Table 4:** Integrated analysis of differences in gene expression and copy number between Proneural GBM and Proneural Oligodendroglioma/Astrocytoma samples.

*Alteration*	*Locus*	*GISTIC q value*	*Gene Symbols*
GBM-Amp	12p13.32	1.21E-03	C1R, C1RL, C1S
GBM-Amp	9p23	1.34E-02	GLIS3, HAUS6
GBM-Amp	9p23	1.34E-02	PLIN2
GBM-Amp	6p21.31	4.30E-02	CLIC1, F13A1
GBM-Amp	6p21.31	4.30E-02	HISTH1C, HISTH2BK, HLA-DQA1, HLA-DQB1, HLA-DRA, HLA-DRB1
OA-Amp	22q11.1	4.69E-04	BCR
OA-Amp	22q11.1	4.69E-04	MN1, RASL10A, SEZ6L
OA-Amp	22q11.1	4.69E-04	SLC25A18
OA-Amp	8q24.22	8.23E-04	FAM84B
OA-Amp	19p13.3	3.10E-03	SHD, SLC1A6
OA-Amp	6p21.2	8.78E-03	DAAM2
OA-Del	19q13.31	1.19E-06	EMP3
OA-Del	4q21.23	8.58E-03	CCDC109B, CFI
OA-Del	4q21.23	8.58E-03	ENPEP, HERC5, IBSP, MLF1IP, SEC24D, SPP1, TDO2

Loci with amplification or deletion and corresponding changes in gene expression in Proneural GBM or Proneural Oligodendrogliomas/Astrocytomas are shown.

Secondly, to gain insight into differences in histologic differentiation and survival, we also performed an integrated analysis of DNA copy number and mRNA gene expression between PN-Oligo samples and PN-GBM samples in the Rembrandt dataset. We observed 323 probe sets corresponding to 240 genes that showed significant amplification or deletion, as well as significant differences in gene expression between these subtypes. A comprehensive list of the mRNA probes that intersect with GISTIC copy number analysis is given in Table S9. Among these genes, of particular interest, were CD44 (amplified at 11p13 and increased in PN-GBM), CDKN2C and CDC42 (both amplified in PN-GBM and deleted in PN-Oligo subtypes at 1p36), TGFB2, TNC, AURKA, and VEGFA, all amplified in PN-GBM, and SOX8 and Noggin (NOG) amplified in PN-Oligo samples.

### Pathway Analysis

**Figure 3:**
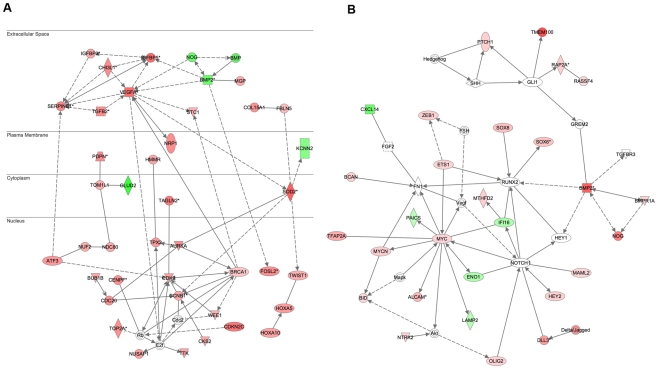
Network analysis of genes differentially expressed between low- and high-grade proneural gliomas identifies regulatory hubs. (**A**) Protein interaction network generated by Ingenuity Pathway Analysis (IPA) of the 576 probes differentially expressed between Proneural GBM and Proneural Oligodendrogliomas/Astrocytomas. Proteins marked in red are increased in Proneural GBM samples, and those in green are decreased, relative to Proneural low grade gliomas. Solid lines represent direct interactions, and dashed lines represent indirect interactions. All interactions are derived on the Ingenuity Knowledgebase, which is extracted from the PubMed literature. (**B**) Protein interaction network based on IPA analysis of 129 genes differentially expressed between PN-Oligodendrogliomas and Classic and Neural Oligodendrogliomas. Network hubs are readily discernible around the Notch receptor, Myc oncogene, and the GLI1 transcription factor.

We performed four separate types of pathway analysis using gene set enrichment analysis (GSEA), the Database for Annotation, Visualization and Integrated Discovery (DAVID), Literature Lab Analysis (LLA), and Ingenuity Pathway Analysis (IPA). The comparison of PN-GBM to PN-OA subtypes identified a number of gene ontologies and pathways that were significantly enriched in multiple analyses (**Table 5**). Pathways, ontologies, and biological processes that were differential between the PN-GBM and PN-OA subtypes included several annotations associated with increased vasculature, including blood coagulation, the complement cascade, vasculature development, wound healing, immune response, and the IKK-NFκB pathway. In addition, IPA identified significant overrepresentation of genes associated with cancer, cell death, proliferation, glioblastomas, and astrocytomas. An overview of gene expression differences between PN-OA and PN-GBM samples is shown in a network diagram (**Figure 3A**) in which genes increased in PN-GBM relative to PN-OA are red, and genes decreased in PN-GBM relative to PN-OA are green. LLA of this gene set identified the ‘Thrombospondin-1 Induced Apoptosis in Microvascular Endothelial Cells’ pathway as the most strongly associated (p = 0.0009) with the differences between PN-GBM and PN-OA samples.

**Table 5:** Significant Gene Ontology and Pathway Annotations identified for 576 probes corresponding to 395 unique genes differentially expressed between Proneural Oligodendrogliomas/Astrocytomas and Proneural GBM.

	*DAVID*	*DAVID*	*GSEA*	*GSEA*	*IPA*	*IPA*
*GO Term*	*Count*	*FDR*	*Count*	*FDR*	*Count*	*p-value*
Response To Wounding	47	1.89E-13			11	5.97E-05
Collagen Fibril Organization	14	1.02E-11			10	1.88E-10
Extracellular Matrix Organization	19	8.33E-09	23	0.16288991	3	1.86E-03
Skeletal System Development	29	2.58E-07			30	1.59E-09
Inflammatory Response	28	1.90E-06			120	1.37E-06
Cell Adhesion	40	3.90E-05			43	1.11E-05
Vasculature Development	21	5.88E-04			16	7.30E-09
Regulation Of Cell Proliferation	38	0.00586091			109	6.41E-11
ECM_Receptor_Interaction			79	0.0247256		
Cell_Communication			107	0.06453185		
Blood_Coagulation			38	0.20890287		
Regulation_Of_MAPKKK_Cascade			18	0.14662187		
Cancer					166	1.78E-27
Apoptosis					61	2.54E-10
Development					30	1.59E-09
Invasion					37	1.75E-09
Brain Cancer					25	4.04E-08
Cell Cycle Progression					51	5.69E-08
Mitosis					29	1.12E-06
Glioma					13	3.05E-05
Astrocytoma					8	4.23E-04

Proneural GBM samples have increased expression of genes associated with enhanced vascularization, proliferation, invasion, and inflammation. Count = number of genes identified with each annotation by the three analyses. FDR = false discovery rate. P-value for IPA is based on the hypergeometric distribution.

In addition, to investigate molecular mechanisms underlying improved survival of the PN-Oligo subtype relative to other types of oligodendrogliomas, we performed pathway analysis of the 129 genes corresponding to the 190 probe sets differentially expressed between PN-Oligo and the Classic and Neural Oligodendrogliomas. As expected, the differential genes were indicative of the proneural subtype, with some of the most significant annotations including Differentiation of Astrocytes, Neurogenesis, Cell Death, Development of Blood Vessels, and Benign Tumor (Table S10). A Network analysis of this set of 129 genes revealed several hubs including GLI1, RUNX2, MYC, BMP2, and NOTCH1 (**Figure 3B**). These data suggest that differences in signaling in the Hedgehog and Notch pathways likely play an important role in the improved outcomes of PN-Oligo cases. Moreover, Literature Lab analysis of the gene sets derived from the integrated expression and copy number analysis comparing PN-Oligo to PN-GBM identified both the Notch (p = 0.0098) and Hedgehog (p = 0.0434) pathways as significantly associated with progression of the proneural subtype.

## Discussion

Here we demonstrate that the Proneural gene expression signature as defined by TCGA is enriched in gliomas with oligodendrogliomal differentiation. This signature also predicts improved outcome for oligodendrogliomas. Analysis of the copy number data from the Rembrandt dataset demonstrated a high frequency of large losses of chromosomes 1p and 19q in oligodendrogliomas, but not in astrocytomas or GBMs. Twelve of the 42 oligodendrogliomas showed co-deletion of at least 85% of chromosomes 1p and 19q, and eleven of those twelve samples were PN-oligos. The other sample with co-deletion of 1p/19q was a classic oligodendroglioma. Nevertheless, our findings regarding the improved outcome of proneural subtype remained significant in multivariate analyses controlling for 1p/19q status and age at diagnosis. Multivariate analysis also showed that the proneural subtype of astroctyoma has significantly better outcome than the classic subtype of astrocytoma, suggesting that the proneural gene expression signature carries prognostic significance across histologic types in the diffuse gliomas.

Our integrated analysis of expression patterns and DNA copy number of low-grade and high-grade proneural gliomas in the Rembrandt dataset identified genes and pathways associated with progression of this disease. Genes increased in high-grade proneural GBMs (PN-GBM) at both the DNA and RNA level included GLIS3, TGFB2, TNC, AURKA, and VEGFA. Network analysis of these gene expression changes identified several regulatory hubs, including GLI1, RUNX2, MYC, BMP2, and NOTCH1. The GLI transcription factors are effectors of the Hedgehog pathway and have been strongly implicated as key regulators of glioblastoma behavior since their discovery. GLI1 regulates stem cell renewal and tumorigenicity of gliomas [23], and targeted inhibition of the Hedgehog pathway results in partial tumor regression in animal models [24]. Tenascin-C (TNC) is induced by TGFβ signaling [25], [26], and promotes invasion of glioma cells through matrix metalloproteinase 12 [27].

Both Ingenuity Pathway Analysis and Literature Lab Analysis identified the Notch pathway as being differentially regulated in low grade and high-grade proneural gliomas. Several components of the Notch pathway, including DLL3 and HEY2 are reduced in PN-GBM, while NOV/CCN3, which is associated with Notch inhibition [28], [29] was increased. These data suggest that Notch pathway activity decreases as proneural gliomas progress, which could account for the abnormal angiogenesis observed in high-grade gliomas, since Notch activity is essential for coordination with VEGF-stimulated sprouting [30]. We also observed several genes associated with angiogenesis and oxidative stress in the analysis of low and high-grade proneural gliomas. These genes included thrombospondin-1 (THBS1), vascular endothelial growth factor A (VEGFA), angiopoietin 2 (ANGPT2), and mitochondrial superoxide dismutase 2 (SOD2). Finally, we observed reduced expression of the pro-apoptotic Bcl2 family member, BH3 interacting domain death agonist (BID) in high-grade proneural gliomas, suggesting that loss of BID expression promotes survival of these high-grade tumors.

As gliomas progress from lower grade (grades II and III) to GBM, hypoxia and necrosis develop centrally, while angiogenesis emerges peripherally. These processes are related, with hypoxia-inducible factors (e.g. VEGFA) secreted by hypoxic, perinecrotic tumor cells, resulting in the development of new vessels. It has been suggested that vascular pathology, including vascular endothelial apoptosis, vascular occlusion and thrombosis, initiates the development of central hypoxia and necrosis. Angiopoetin 2 has been implicated in initiating endothelial apoptosis by Tie2 receptor in this setting [31], [32], [33]. THBS-1, a protein that inhibits angiogenesis by inducing apoptosis via activation of CD36 in microvascular endothelial cells, may be relevant to these mechanisms as well, as we found this pathway to be markedly upregulated in GBMs compared to lower grade gliomas. The non-receptor tyrosine kinase FYN is activated by THBS-1 through CD36, activating the apoptosis inducing proteases like caspase-3 and p38 protein kinases. p38 is a mitogen-activated kinase that also induces apoptosis in some conditions, perhaps through AP-1 activation and the activation of genes that lead to apoptosis.

In summary, our *in silico* analysis of available molecular datasets for low and high-grade gliomas has provided new insights into the survival and progression of the proneural subtype. These findings demonstrate the power and importance of publicly available molecular datasets and the potential for future discoveries from The Cancer Genome Atlas project.

## Supporting Information

Table S1 Complete clinical data downloaded from the Rembrandt public data repository (https://caintegrator.nci.nih.gov/rembrandt/) using data available on November 11, 2009. Clinical data were derived from contributing center institutional diagnoses at Henry Ford Hospital, UCSF, Lee Moffitt Cancer Center, Dana Farber Cancer Center, University of Wisconsin, and NCI.
(0.15 MB XLS)

Table S2 Survival and PAM subtype classification data for Rembrandt samples used in the survival analysis.
(0.05 MB PDF)

Table S3 Complete Comparative Marker Selection Results generated from Rembrandt data comparing low- and high-grade proneural samples. Data were generated using GenePattern 3.0.
(7.50 MB TXT)

Table S4 Complete Comparative Marker Selection Results generated from Rembrandt data comparing proneural oligodendroglioma and proneural GBM samples. Data were generated using GenePattern 3.0.
(7.50 MB TXT)

Table S5 Complete Comparative Marker Selection Results generated from Rembrandt data comparing proneural oligodendroglioma and neural oligodendroglioma samples. Data were generated using GenePattern 3.0.
(7.50 MB TXT)

Table S6 Complete Comparative Marker Selection Results generated from Rembrandt data comparing proneural oligodendroglioma and classic oligodendroglioma samples. Data were generated using GenePattern 3.0.
(7.50 MB TXT)

Table S7 Significant Comparative Marker Selection Results generated from Rembrandt data comparing proneural oligodendroglioma and all other oligodendroglioma samples. Data were generated using GenePattern 3.0.
(0.15 MB XLS)

Table S8 Integrated RNA and DNA Copy number analysis comparison of low- and high-grade proneural gliomas.
(0.19 MB XLS)

Table S9 Integrated RNA and DNA Copy number analysis comparison of proneural oligodendrogliomas and proneural GBM samples.
(0.15 MB XLS)

Table S10 Gene Ontology analysis of the 129 genes differentially expressed between proneural oligodendrogliomas and the all other oligodendrogliomas.
(0.02 MB XLS)

Figure S1 Kaplan-Meier Analysis of Low-grade gliomas.
(0.13 MB TIF)
