# Supplementary material for: The Proneural Molecular Signature Is Enriched in Oligodendrogliomas and Predicts Improved Survival among Diffuse Gliomas
Source: PLoS One. 2010 Sep 3;5(9):e12548. doi: 10.1371/journal.pone.0012548 (PMC2933229; doi:10.1371/journal.pone.0012548)
Supplement: Table S2 — Survival and PAM subtype classification data for Rembrandt samples used in the survival analysis. (0.05 MB PDF) [file pone.0012548.s002.pdf]

name	time	status	cls.PAM	Disease	Grade	Age at Dx (years)	1p/19q deleted
HF1232	21	1	Classic	ASTROCYTOMA	III	50-54	No
HF1139	15	1	Classic	ASTROCYTOMA	III	40-44	No
E09826	21	1	Classic	ASTROCYTOMA	--	40-44	No
E09334	60	0	Mesenchymal	ASTROCYTOMA	--	40-44	N.D.
E09800	21	1	Mesenchymal	ASTROCYTOMA	--	65-69	No
E10003	11	1	Mesenchymal	ASTROCYTOMA	--	60-64	No
HF0757	60	0	Mesenchymal	ASTROCYTOMA	II	40-44	No
HF1246	1	1	Mesenchymal	ASTROCYTOMA	II	65-69	No
HF0450	27	1	Neural	ASTROCYTOMA	III	30-34	No
E09471	27	1	Neural	ASTROCYTOMA	--	70-74	No
HF1708	60	0	Neural	ASTROCYTOMA	II	20-24	No
HF0223	45	1	Neural	ASTROCYTOMA	III	45-49	No
HF1032	27	1	Neural	ASTROCYTOMA	III	40-44	No
HF1469	21	1	Neural	ASTROCYTOMA	III	45-49	No
HF0778	8	1	Neural	ASTROCYTOMA	III	65-69	No
HF1581	1	1	Neural	ASTROCYTOMA	III	65-69	No
HF1407	6	1	Proneural	ASTROCYTOMA	III	55-59	No
HF0152	60	0	Proneural	ASTROCYTOMA	III	30-34	No
HF1487	11	1	Proneural	ASTROCYTOMA	III	60-64	No
E09394	54	1	Proneural	ASTROCYTOMA	--	35-39	N.D.
E07733	54	1	Proneural	ASTROCYTOMA	--	50-54	No
HF1511	54	1	Proneural	ASTROCYTOMA	II	25-29	No
HF1442	45	1	Proneural	ASTROCYTOMA	II	35-39	No
HF1568	39	1	Proneural	ASTROCYTOMA	II	30-34	No
HF0108	60	0	Proneural	ASTROCYTOMA	III	35-39	No
HF1587	60	0	Proneural	ASTROCYTOMA	III	30-34	N.D.
HF0017	60	0	Proneural	ASTROCYTOMA	III	45-49	No
HF0026	54	1	Proneural	ASTROCYTOMA	III	60-64	No
HF1000	39	1	Proneural	ASTROCYTOMA	III	30-34	No
HF1295	21	1	Proneural	ASTROCYTOMA	III	55-59	No
HF1269	15	1	Proneural	ASTROCYTOMA	III	45-49	No
HF1344	8	1	Proneural	ASTROCYTOMA	III	60-64	No
E09833	21	1	Classic	GBM	IV	50-54	No
E09167	15	1	Classic	GBM	IV	65-69	No
E09704	11	1	Classic	GBM	IV	50-54	N.D.
E09774	8	1	Classic	GBM	IV	50-54	No
HF0990	60	0	Classic	GBM	IV	40-44	No
HF1077	60	0	Classic	GBM	IV	40-44	No
HF1382	45	1	Classic	GBM	IV	60-64	No
HF1057	27	1	Classic	GBM	IV	60-64	No
HF1628	15	1	Classic	GBM	IV	45-49	No
HF0408	15	1	Classic	GBM	IV	55-59	No
HF0894	15	1	Classic	GBM	IV	55-59	No
HF0184	11	1	Classic	GBM	IV	50-54	No
HF0066	11	1	Classic	GBM	IV	55-59	No
HF1540	11	1	Classic	GBM	IV	75-79	No
HF0627	11	1	Classic	GBM	IV	45-49	No
HF1517	8	1	Classic	GBM	IV	55-59	No
HF0702	8	1	Classic	GBM	IV	50-54	No
HF1608	8	1	Classic	GBM	IV	50-54	No
HF1458	8	1	Classic	GBM	IV	45-49	No
HF1490	4	1	Classic	GBM	IV	65-69	No
HF1589	4	1	Classic	GBM	IV	65-69	No
HF1618	3	1	Classic	GBM	IV	50-54	No
HF1509	3	1	Classic	GBM	IV	55-59	N.D.
HF0138	2	1	Classic	GBM	IV	75-79	No
HF0142	1	1	Classic	GBM	IV	70-74	N.D.
HF1191	1	1	Classic	GBM	IV	70-74	No
E09791	15	1	Classic	GBM	IV	55-59	No
HF0316	60	0	Classic	GBM	IV	40-44	N.D.
HF0986	60	0	Classic	GBM	IV	35-39	No
HF0445	45	1	Classic	GBM	IV	30-34	No
HF1397	27	1	Classic	GBM	IV	60-64	No
HF1262	21	1	Classic	GBM	IV	55-59	No
HF1137	21	1	Classic	GBM	IV	70-74	No
HF1078	21	1	Classic	GBM	IV	45-49	N.D.
HF1286	15	1	Classic	GBM	IV	75-79	No
HF1178	15	1	Classic	GBM	IV	70-74	No
HF0460	11	1	Classic	GBM	IV	55-59	No
HF1538	4	1	Classic	GBM	IV	70-74	No
HF1255	4	1	Classic	GBM	IV	45-49	No
E09832	6	1	Classic	GBM	IV	65-69	No
HF0268	21	1	Classic	GBM	IV	45-49	N.D.
HF1409	15	1	Classic	GBM	IV	50-54	No
E09938	27	1	Mesenchymal	GBM	IV	45-49	N.D.
E10016	27	1	Mesenchymal	GBM	IV	50-54	N.D.
E09511	15	1	Mesenchymal	GBM	IV	45-49	No
E09606	15	1	Mesenchymal	GBM	IV	50-54	No
E09649	11	1	Mesenchymal	GBM	IV	45-49	No
E09483	11	1	Mesenchymal	GBM	IV	45-49	No
E09654	8	1	Mesenchymal	GBM	IV	65-69	N.D.
E09451	6	1	Mesenchymal	GBM	IV	65-69	No
E09602	5	1	Mesenchymal	GBM	IV	65-69	No
HF0543	60	0	Mesenchymal	GBM	IV	50-54	No
HF1292	54	1	Mesenchymal	GBM	IV	45-49	N.D.
HF1585	21	1	Mesenchymal	GBM	IV	60-64	No
HF1356	15	1	Mesenchymal	GBM	IV	55-59	No
HF1280	15	1	Mesenchymal	GBM	IV	40-44	No
HF0583	15	1	Mesenchymal	GBM	IV	60-64	No
HF0608	11	1	Mesenchymal	GBM	IV	50-54	No

HF1220	11	1	Mesenchymal	GBM	IV	60-64	No
HF0520	8	1	Mesenchymal	GBM	IV	60-64	No
HF1097	8	1	Mesenchymal	GBM	IV	50-54	No
HF0790	8	1	Mesenchymal	GBM	IV	70-74	No
HF0031	1	1	Mesenchymal	GBM	IV	65-69	No
E09238	21	1	Neural	GBM	IV	45-49	No
HF0050	27	1	Neural	GBM	IV	50-54	No
HF0089	8	1	Neural	GBM	IV	65-69	No
E09531	21	1	Neural	GBM	IV	55-59	No
E09670	15	1	Neural	GBM	IV	70-74	No
HF0048	54	1	Neural	GBM	IV	30-34	N.D.
HF1058	15	1	Neural	GBM	IV	50-54	No
HF1316	8	1	Neural	GBM	IV	70-74	N.D.
HF1640	6	1	Neural	GBM	IV	65-69	No
HF0024	6	1	Neural	GBM	IV	75-79	No
HF0505	4	1	Neural	GBM	IV	75-79	No
HF1667	3	1	Neural	GBM	IV	65-69	No
HF0982	15	1	Proneural	GBM	IV	65-69	No
HF1534	8	1	Proneural	GBM	IV	55-59	No
HF1671	15	1	Proneural	GBM	IV	65-69	No
HF1122	8	1	Proneural	GBM	IV	35-39	No
E09852	45	1	Proneural	GBM	IV	45-49	No
E09965	21	1	Proneural	GBM	IV	40-44	No
HF0244	8	1	Proneural	GBM	IV	60-64	No
HF0936	5	1	Proneural	GBM	IV	55-59	No
E09802	33	1	Proneural	GBM	IV	60-64	No
E09647	21	1	Proneural	GBM	IV	70-74	No
E09998	2	1	Proneural	GBM	IV	60-64	No
HF1242	60	0	Proneural	GBM	IV	35-39	No
HF0996	60	0	Proneural	GBM	IV	50-54	N.D.
HF0435	60	0	Proneural	GBM	IV	40-44	No
HF1494	33	1	Proneural	GBM	IV	30-34	No
HF1475	33	1	Proneural	GBM	IV	15-19	N.D.
HF1357	33	1	Proneural	GBM	IV	20-24	No
HF0992	21	1	Proneural	GBM	IV	75-79	No
HF1186	21	1	Proneural	GBM	IV	30-34	No
HF0855	15	1	Proneural	GBM	IV	55-59	No
HF0654	15	1	Proneural	GBM	IV	55-59	No
HF0963	11	1	Proneural	GBM	IV	75-79	No
HF1318	8	1	Proneural	GBM	IV	50-54	No
HF1338	6	1	Proneural	GBM	IV	50-54	No
HF1702	6	1	Proneural	GBM	IV	75-79	No
HF0180	1	1	Proneural	GBM	IV	70-74	No
E09966	15	1	Classic	OLIGODENDROGLIOMA	--	55-59	No
HF1345	60	0	Classic	OLIGODENDROGLIOMA	II	15-19	No
HF0327	21	1	Classic	OLIGODENDROGLIOMA	II	70-74	N.D.
HF1150	21	1	Classic	OLIGODENDROGLIOMA	III	70-74	No
HF0510	21	1	Classic	OLIGODENDROGLIOMA	III	70-74	No
HF0816	45	1	Classic	OLIGODENDROGLIOMA	III	45-49	Yes
E09513	15	1	Neural	OLIGODENDROGLIOMA	--	50-54	No
E09664	27	1	Neural	OLIGODENDROGLIOMA	--	40-44	No
HF0599	45	1	Neural	OLIGODENDROGLIOMA	II	50-54	No
HF0285	15	1	Neural	OLIGODENDROGLIOMA	II	60-64	No
HF1167	15	1	Neural	OLIGODENDROGLIOMA	II	45-49	No
HF1381	60	0	Proneural	OLIGODENDROGLIOMA	II	25-29	No
HF1502	8	1	Proneural	OLIGODENDROGLIOMA	III	35-39	No
HF0087	60	0	Proneural	OLIGODENDROGLIOMA	--	60-64	Yes
E09448	60	0	Proneural	OLIGODENDROGLIOMA	--	40-44	No
E09959	45	1	Proneural	OLIGODENDROGLIOMA	--	50-54	N.D.
HF1489	60	0	Proneural	OLIGODENDROGLIOMA	II	50-54	No
HF0931	60	0	Proneural	OLIGODENDROGLIOMA	II	35-39	No
HF1677	60	0	Proneural	OLIGODENDROGLIOMA	II	50-54	Yes
HF0962	60	0	Proneural	OLIGODENDROGLIOMA	II	30-34	Yes
HF0899	60	0	Proneural	OLIGODENDROGLIOMA	II	55-59	No
HF0726	60	0	Proneural	OLIGODENDROGLIOMA	II	35-39	N.D.
HF1235	60	0	Proneural	OLIGODENDROGLIOMA	II	35-39	No
HF1551	60	0	Proneural	OLIGODENDROGLIOMA	II	30-34	Yes
HF1613	60	0	Proneural	OLIGODENDROGLIOMA	II	35-39	Yes
HF1606	60	0	Proneural	OLIGODENDROGLIOMA	II	60-64	No
HF0914	60	0	Proneural	OLIGODENDROGLIOMA	II	40-44	No
HF1325	60	0	Proneural	OLIGODENDROGLIOMA	II	35-39	No
HF0670	60	0	Proneural	OLIGODENDROGLIOMA	II	50-54	N.D.
HF0291	60	0	Proneural	OLIGODENDROGLIOMA	II	50-54	No
HF0960	60	0	Proneural	OLIGODENDROGLIOMA	II	30-34	No
HF0835	45	1	Proneural	OLIGODENDROGLIOMA	II	35-39	N.D.
HF0975	39	1	Proneural	OLIGODENDROGLIOMA	II	60-64	Yes
HF0434	6	1	Proneural	OLIGODENDROGLIOMA	II	60-64	Yes
HF0920	1	1	Proneural	OLIGODENDROGLIOMA	II	85-89	N.D.
HF0966	60	0	Proneural	OLIGODENDROGLIOMA	III	50-54	N.D.
HF1156	60	0	Proneural	OLIGODENDROGLIOMA	III	30-34	Yes
HF1227	60	0	Proneural	OLIGODENDROGLIOMA	III	25-29	Yes
HF1334	60	0	Proneural	OLIGODENDROGLIOMA	III	20-24	No
HF1185	60	0	Proneural	OLIGODENDROGLIOMA	III	25-29	No
HF1136	45	1	Proneural	OLIGODENDROGLIOMA	III	35-39	No
HF1493	39	1	Proneural	OLIGODENDROGLIOMA	III	70-74	Yes
HF0251	21	1	Proneural	OLIGODENDROGLIOMA	III	50-54	Yes

Proneural	Neural	Classic	Mesenchymal	PAM-prob-Classic	PAM-prob-Mesenchymal	PAM-prob-Neural	PAM-prob-Proneural
-0.17548331	0.03722061	0.04111276	-0.16002193	0.973601082	2.35E-47	0.026398903	1.56E-08
-0.30727016	-0.21491082	0.22588909	-0.11786833	1	4.20E-63	6.61E-75	1.96E-57
-0.23569581	0.04020046	0.03738289	0.02570715	1	2.32E-36	6.40E-11	2.19E-24
-0.31123483	-0.03509213	-0.10033232	0.2409136	8.14E-25	1	1.66E-16	3.26E-47
-0.27750141	-0.09960012	0.01246473	0.37678394	1.44E-66	1	1.64E-111	2.27E-132
-0.26556256	-0.09405149	-0.04003804	0.40628655	9.16E-116	1	1.82E-164	4.01E-195
-0.02834075	0.00940329	-0.01582836	0.24114743	2.53E-27	1	1.35E-25	2.12E-35
-0.10851956	0.10600719	-0.12014526	0.3657104	9.70E-58	1	2.41E-24	2.50E-62
-0.08162595	0.01977534	-0.12230225	0.12504465	2.35E-19	4.37E-17	1	4.00E-10
-0.08501583	0.32200499	-0.10386991	0.03805214	2.23E-38	6.76E-56	1	4.35E-31
-0.02177614	0.26468447	0.01589119	-0.09905855	1.40E-31	5.62E-83	1	8.42E-22
0.05663593	0.25128685	-0.10714295	-0.03736741	5.76E-33	1.08E-79	0.999999015	9.85E-07
0.20653827	0.31238275	-0.10150528	-0.03119725	6.74E-55	1.24E-95	0.999999984	1.59E-08
0.01231168	0.30892749	-0.00031496	-0.12633369	1.50E-17	3.42E-72	1	7.25E-13
0.03620391	0.13226847	-0.10416799	-0.0703419	1.72E-43	2.59E-75	0.999998698	1.30E-06
-0.11256031	0.22326553	0.02662414	-0.20391371	7.74E-18	1.85E-86	0.999999991	8.90E-09
0.02665198	0.02440749	-0.01418338	0.12885957	7.79E-27	3.99E-35	8.24E-22	1
0.1455686	0.29754711	-0.07074295	-0.20408589	1.33E-54	1.65E-123	1.54E-18	1
-0.01739361	0.05984597	-0.06411859	-0.14283065	8.92E-43	1.55E-93	3.05E-16	1
0.26500441	-0.03616724	-0.05167829	-0.27476351	4.81E-103	1.75E-192	5.91E-92	1
0.34465298	0.12624758	-0.03545295	-0.15727696	3.16E-74	1.44E-135	2.81E-53	1
0.2685822	0.3154403	-0.07882809	-0.16596784	4.56E-65	4.27E-124	1.02E-21	1
0.29244119	0.18590577	0.0154343	-0.17562899	2.06E-69	4.27E-145	6.62E-51	1
0.26917586	0.08192786	-0.02309892	-0.1645396	3.67E-66	2.27E-124	1.53E-46	1
0.41185962	-0.06555543	-0.05731561	-0.15756578	3.05E-117	1.12E-196	2.34E-121	1
0.3259946	0.19882599	-0.12011051	-0.23815858	6.51E-91	5.65E-167	9.08E-55	1
0.21282139	0.01918477	-0.04828767	-0.03874205	7.74E-52	2.86E-103	3.17E-46	1
0.28920027	0.26260255	-0.09530166	-0.154178	5.12E-68	3.04E-127	1.67E-28	1
0.40024935	0.03957784	-0.04370796	-0.15994365	1.77E-104	3.78E-180	1.04E-95	1
0.32946307	0.0939639	-0.11177817	-0.11588081	3.39E-69	1.66E-111	5.42E-38	1
0.22114941	0.00769947	-0.1243125	0.00226955	1.19E-43	1.70E-54	2.11E-35	1
0.11587421	-0.08773361	0.05883813	-0.27359261	2.81E-23	1.10E-119	1.26E-65	1
-0.39943747	-0.15312173	0.10588549	-0.10354404	1	1.10E-53	8.63E-56	7.52E-57
-0.38772947	-0.16391182	0.18835137	-0.01496568	1	4.16E-57	4.54E-87	1.57E-92
-0.24452018	-0.08483984	0.07459009	-0.10188429	1	1.19E-51	7.02E-26	5.52E-25
-0.2742313	-0.10306595	0.12827172	0.03114713	1	1.16E-38	4.17E-44	1.72E-51
-0.07342741	-0.13760946	0.24255159	-0.0717974	1	4.11E-96	5.66E-77	2.26E-23
-0.30402531	-0.1157238	0.16257148	-0.009684	1	1.14E-29	3.01E-47	4.62E-49
-0.04541159	-0.07212039	0.18913044	-0.22834151	1	3.37E-91	4.19E-63	1.11E-23
-0.12185591	-0.09910027	0.16170549	-0.08660006	0.999992492	1.03E-59	2.52E-37	7.51E-06
-0.19967819	-0.21478497	0.29476509	-0.19408718	1	5.10E-119	1.35E-120	8.56E-61
-0.35437379	-0.16974918	0.1200068	-0.06173281	1	5.39E-49	4.88E-57	1.95E-57
-0.41035484	-0.25491013	0.10969371	0.01174549	1	2.15E-32	1.65E-81	1.09E-69
-0.31501842	-0.17031877	0.29982508	-0.10642419	1	4.95E-92	1.94E-105	3.66E-95
-0.21434355	-0.18979278	0.25220394	-0.14638042	1	2.24E-89	4.87E-102	2.00E-66
-0.37820088	-0.25029108	0.17645416	0.00778511	1	7.51E-33	2.46E-86	3.89E-88
-0.24015677	-0.09821462	0.14794859	0.03151229	1	1.69E-29	6.98E-58	5.34E-55
-0.34346515	-0.13807583	0.24928976	-0.10210694	1	9.48E-68	3.05E-81	3.83E-76
-0.14161178	0.03011735	0.17684226	-0.20429304	1	1.99E-98	6.22E-44	8.86E-24
-0.28887407	-0.25940619	0.15691449	-0.02788222	1	1.02E-50	6.91E-85	2.06E-56
-0.32692948	-0.25153413	0.1554062	-0.08613926	1	5.17E-63	4.18E-78	8.16E-61
-0.36087857	-0.15095694	0.25588424	-0.15837012	1	1.94E-87	6.58E-89	2.21E-88
-0.4607918	-0.1992977	0.16785498	0.02584397	1	3.57E-37	2.88E-88	1.11E-102
-0.38973597	-0.23392828	0.22067026	-0.06335671	1	1.76E-67	5.82E-110	1.61E-102
-0.36882161	-0.12401431	0.10290742	-0.03961462	1	2.61E-27	2.25E-42	1.78E-62
-0.2995607	-0.03092742	0.04656936	-0.06916207	1	6.92E-39	4.57E-25	3.96E-28
-0.22736428	-0.2116593	0.25694975	-0.16144594	1	1.12E-91	5.41E-109	3.55E-65
-0.3174331	-0.17962659	0.2334104	-0.08287329	1	2.27E-68	1.96E-74	1.85E-65
-0.30603324	-0.00121195	0.00178055	0.00631502	1	8.47E-27	8.29E-17	1.12E-40
-0.21383546	0.04653945	0.08846691	0.0889534	1	9.26E-22	3.44E-14	7.68E-24
-0.16997582	-0.16047248	0.01297989	0.04109141	1	1.49E-32	1.47E-51	1.21E-47
0.03931097	-0.00697185	-0.02218171	0.16757862	0.999999992	6.26E-09	1.76E-13	1.55E-09
-0.30090525	-0.15581483	0.02864312	0.08801564	0.999999995	5.25E-09	9.81E-52	1.51E-44
-0.3828268	-0.14970652	0.06797526	0.19375123	0.999999995	5.30E-09	7.45E-56	2.27E-62
-0.378276	-0.18899162	0.1045713	0.14712171	0.782808311	0.217191689	5.39E-69	8.54E-85
-0.17429013	-0.15576975	0.01590906	0.1319131	0.999998305	1.70E-06	1.24E-54	3.04E-33
-0.37650087	-0.13142243	0.08690157	0.1925082	0.999968936	3.11E-05	3.18E-68	3.66E-94
-0.23865017	-0.10373047	0.00966826	0.02371736	1	1.67E-22	1.86E-27	8.70E-11
-0.38314827	-0.11926156	0.03285557	0.25482086	0.806032422	0.193967578	1.58E-49	7.87E-68
-0.26134692	-0.15213704	0.03401277	0.19159751	0.999999845	1.55E-07	8.58E-40	1.22E-34
-0.25418725	-0.01319754	0.04914334	0.07544105	0.55861158	0.44003312	0.001355299	6.92E-14
-0.25066755	0.06802561	0.03281914	-0.06440237	1	1.72E-43	3.73E-17	3.08E-30
-0.20136507	0.22353612	0.04650536	-0.05130934	0.616291537	5.58E-50	0.383708463	7.63E-32
-0.19963801	0.0097544	-0.03415866	0.0003145	0.999999996	1.55E-18	4.41E-09	3.52E-27
-0.13555625	0.00463045	-0.05081707	0.41965459	1.13E-65	1	2.43E-74	1.06E-98
-0.25687156	-0.085568	-0.0750297	0.2837869	3.42E-62	1	2.38E-65	3.15E-101
-0.1434922	-0.05300305	-0.05003812	0.43917289	7.10E-77	1	1.44E-86	4.77E-102
-0.22493065	-0.02574323	-0.1172613	0.37806405	1.15E-80	1	1.96E-66	2.13E-103
-0.15518661	0.13235542	-0.1277981	0.31816973	1.95E-13	0.993172772	0.006827228	1.46E-18
-0.35450525	-0.0865225	0.04973416	0.2874398	1.01E-37	1	2.27E-84	2.12E-117
-0.37746284	-0.09152878	-0.00022567	0.25010017	3.79E-07	0.999999621	2.18E-32	5.48E-52
-0.28401706	-0.16627996	0.01401749	0.21082045	3.16E-18	1	4.11E-77	1.71E-65
-0.31105817	-0.00718174	-0.04981443	0.27255515	1.78E-21	1	4.08E-37	1.66E-63
-0.33776117	-0.12349815	-0.03428909	0.32925111	1.16E-76	1	2.25E-129	1.19E-153
-0.2900596	-0.12297064	0.034008	0.30012238	3.69E-38	1	9.96E-102	2.99E-112
-0.17533869	0.04276176	-0.07199567	0.26929604	8.93E-13	1	3.50E-18	4.26E-26
-0.16907479	-0.02131682	-0.06619232	0.38568561	1.09E-53	1	1.17E-66	9.58E-78
-0.28626911	-0.07399953	-0.00291189	0.16963521	1.55E-23	1	1.48E-55	1.38E-77
-0.29883143	-0.02893055	-0.03795069	0.14294972	4.19E-05	0.999958111	1.52E-15	7.92E-34
-0.25028244	0.10375744	-0.0842127	0.28914348	4.10E-21	0.999991172	8.83E-06	5.37E-46

-0.30235288	-0.01613086	-0.0244793	0.27617447	5.33E-32	1	6.73E-63	8.12E-89
-0.28401688	-0.00872185	-0.06266611	0.35977284	3.20E-44	1	7.61E-54	8.86E-85
-0.14589056	0.00140202	0.03794677	0.25273671	1.07E-48	1	2.96E-89	1.02E-99
-0.36594416	-0.17954004	0.05129005	0.22400734	2.14E-09	0.999999998	8.69E-81	7.22E-91
-0.33587359	-0.13641375	-0.00162913	0.28144684	3.81E-32	1	1.23E-76	7.32E-93
0.00324426	0.05232273	-0.12694784	0.17088045	2.16E-43	3.38E-23	1	1.91E-15
-0.12006583	0.05458275	-0.15014483	0.21174128	3.96E-26	0.181972775	0.818027225	5.82E-28
-0.20003401	0.00619971	-0.12398337	0.12289294	3.97E-17	1.39E-08	0.999999986	3.85E-21
0.04162941	0.29012603	-0.08807226	0.02602697	8.26E-41	4.06E-68	1	5.56E-14
0.13033656	0.38972338	-0.10391602	-0.07201977	5.30E-65	6.13E-107	1	2.82E-17
0.02371049	0.2626009	-0.09486119	0.05102597	1.98E-30	4.71E-64	1	6.60E-17
0.20187064	0.3543242	-0.10479236	-0.0787432	3.02E-60	3.08E-107	0.999999612	3.88E-07
-0.23042139	0.19953903	0.01634551	0.09503303	5.65E-21	1.31E-32	1	1.45E-36
0.16991298	0.324886	-0.06142996	-0.07308323	1.18E-57	5.24E-113	0.833619243	0.166380757
-0.15295024	0.06659551	-0.14905112	0.04470661	0.000346318	2.12E-21	0.866987365	0.132666317
0.16703054	0.32616193	-0.13239868	-0.07264175	1.65E-60	1.25E-104	1	2.53E-10
0.09307416	0.28193236	-0.12256395	-0.12606261	2.03E-54	7.00E-101	0.999999998	1.94E-09
-0.02758078	-0.09959868	0.03844924	-0.05642089	0.003628588	4.65E-45	1.11E-21	0.996371412
-0.15747282	-0.09084948	-0.01662733	-0.09127501	0.015438516	9.28E-47	1.22E-21	0.984561484
-0.04007235	-0.12563729	0.01490673	0.04570972	0.003833072	3.21E-41	7.30E-29	0.996166928
0.05267777	-0.19088033	0.0369162	0.06051635	0.000400102	6.31E-34	2.18E-71	0.999599898
0.12201058	0.1761939	-0.01117324	-0.11019316	8.88E-43	8.87E-108	1.11E-24	1
0.03910664	0.09490998	-0.10261252	-0.00940163	3.60E-26	6.47E-64	2.97E-12	1
0.13239279	0.1697304	-0.02283916	-0.04693899	4.40E-27	3.03E-80	2.45E-25	1
0.07640625	0.24767957	-0.15407635	0.15815959	9.45E-24	2.73E-22	0.155704913	0.844295087
0.39687026	-0.07771062	-0.00635114	-0.09977523	2.78E-57	6.50E-103	2.86E-71	1
0.40691201	0.01193259	-0.0404053	-0.02352294	4.78E-62	2.57E-93	7.96E-65	1
0.21474639	-0.15289309	-0.04719067	-0.06062698	7.42E-52	1.76E-93	2.09E-79	1
0.29250415	-0.09404856	-0.04643922	0.00862008	9.65E-66	3.54E-97	2.14E-70	1
0.23594349	-0.19761236	-0.10767816	-0.11015224	5.44E-48	2.44E-114	1.37E-93	1
0.1715094	-0.01740279	-0.01765762	-0.09722135	1.51E-35	3.22E-89	1.97E-43	1
0.36139893	-0.07441909	0.0155358	-0.04695105	1.51E-70	3.56E-122	2.87E-96	1
0.28493828	0.0540374	-0.03180342	-0.18232999	3.65E-60	1.05E-126	2.14E-62	1
0.16426384	0.11461577	-0.04672759	-0.01794303	6.26E-33	9.28E-71	5.88E-15	1
0.25004618	-0.14451204	-0.0260565	0.03214212	2.35E-24	4.45E-55	2.05E-53	1
0.24074345	-0.10170515	-0.04838877	-0.0419252	1.32E-54	7.93E-94	1.88E-62	1
0.43317008	-0.04742233	-0.06800764	-0.15942032	6.38E-90	1.60E-149	2.87E-89	1
0.33903631	-0.10866722	-0.02211334	0.01479992	6.06E-57	2.26E-83	1.67E-60	1
0.20190506	-0.1222316	0.00332991	-0.07043159	1.44E-35	9.71E-71	4.93E-53	1
0.33529984	-0.12864297	-0.05495747	0.15786664	8.41E-39	8.65E-54	4.33E-60	1
0.33195129	-0.02826449	-0.04350018	-0.21959331	7.32E-62	2.24E-133	1.26E-69	1
0.32865715	-0.06844907	-0.02599935	0.04562388	1.75E-61	3.45E-88	3.79E-68	1
0.35676814	-0.07356744	-0.06023162	-0.06743856	2.84E-85	5.35E-127	1.23E-99	1
-0.16769663	-0.2023638	0.14865411	-0.17742386	1	2.49E-94	4.97E-73	5.84E-34
0.05939938	0.02173562	0.11496749	-0.02906838	0.999996933	8.73E-58	1.35E-22	3.07E-06
-0.14570919	-0.10362383	0.05509786	-0.03128611	0.999881953	3.92E-39	1.03E-46	0.000118047
-0.13953337	-0.1528541	0.20958763	-0.12918198	1	8.51E-84	1.68E-58	1.37E-22
-0.29968743	-0.04101851	0.031759	-0.02175229	1	6.71E-30	2.01E-28	1.25E-21
-0.16271534	0.08451829	0.04438126	-0.11132914	0.99997469	1.95E-43	2.53E-05	2.72E-14
0.03388568	0.0985932	-0.0594822	0.14788595	3.81E-20	3.15E-30	1	1.37E-14
-0.01284146	0.17789807	-0.11481051	0.07798261	1.39E-37	2.47E-53	0.999998765	1.24E-06
0.20046634	0.30094199	-0.10090303	-0.00377117	5.06E-60	5.53E-94	0.999999996	4.12E-09
0.17436055	0.34682044	-0.13730476	-0.14147308	2.25E-77	7.42E-125	0.999999997	3.40E-09
-0.18391284	0.08212059	0.0606458	0.04393613	0.054666623	4.18E-39	0.945333377	8.91E-19
0.11941066	0.18175713	-0.00689565	-0.13903434	1.70E-49	6.64E-110	2.38E-31	1
0.20509046	0.36501178	-0.083884	-0.11779613	3.20E-62	1.36E-123	6.60E-08	0.999999934
0.43395896	0.03190222	-0.10555529	-0.2430786	3.26E-102	1.14E-182	7.61E-92	1
0.41648301	-0.07131375	0.00137297	-0.06762	2.12E-59	1.96E-141	9.23E-103	1
0.44042789	0.10246982	-0.01727187	-0.16219116	2.92E-95	4.10E-167	1.81E-85	1
0.42653873	0.03532591	-0.09554082	-0.24422538	2.02E-103	1.60E-184	9.50E-92	1
0.40342675	0.01181073	-0.05007529	-0.20637034	3.90E-99	1.43E-168	1.42E-87	1
0.40007119	0.08589984	-0.12771566	-0.20757812	6.52E-96	5.48E-167	4.36E-67	1
0.38502411	0.01236302	-0.07937852	-0.19914141	1.35E-102	3.56E-185	1.56E-96	1
0.37343931	0.15636191	-0.09315176	-0.16859879	1.00E-77	3.07E-140	4.03E-50	1
0.3716583	0.05203333	-0.07898084	-0.17856516	2.84E-84	6.94E-155	2.19E-74	1
0.36823895	-0.01923605	-0.07213565	-0.23556927	7.64E-106	3.80E-195	6.00E-104	1
0.36414263	0.04560779	-0.07091859	-0.23921573	1.27E-85	2.19E-161	9.73E-77	1
0.3399543	0.02349958	-0.03563984	-0.22791696	1.58E-96	1.11E-173	1.82E-85	1
0.32870745	0.08098469	-0.03147051	-0.14879582	5.10E-71	9.89E-132	1.02E-61	1
0.31227039	0.0050992	-0.08221999	-0.02177863	1.72E-73	2.59E-108	6.61E-57	1
0.3013896	-0.05693597	-0.02110722	-0.21072639	2.88E-102	3.41E-183	2.14E-100	1
0.22695215	0.01419946	-0.02764897	-0.16778842	1.14E-66	3.94E-132	6.49E-64	1
0.22669438	0.10118835	-0.06927998	-0.21899866	1.40E-73	6.94E-141	2.33E-54	1
0.22007993	0.05173498	-0.03851671	-0.19286769	9.67E-64	1.35E-133	5.69E-48	1
0.29732113	0.10337098	-0.0531218	-0.19848639	2.11E-77	3.71E-153	1.24E-66	1
0.41163669	0.0550953	-0.08915083	-0.20382258	4.19E-107	1.43E-186	6.80E-86	1
0.32081461	0.10420607	-0.13475782	-0.20002437	1.16E-82	1.30E-149	3.40E-61	1
0.36896782	-0.0312714	-0.12586676	-0.13818005	1.06E-103	2.88E-177	5.74E-101	1
0.37389313	0.02446722	-0.01757404	-0.10291857	1.09E-105	5.65E-159	7.45E-86	1
0.35072244	-0.01214959	-0.03272106	-0.21112607	1.24E-88	2.59E-167	3.27E-85	1
0.31024662	0.1590435	-0.10792608	-0.21416702	6.50E-75	9.98E-139	5.70E-49	1
0.29401963	-0.0114594	-0.04599502	-0.04345158	6.01E-61	7.99E-118	9.80E-66	1
0.03919209	0.02308593	0.03062321	-0.05492026	1.26E-12	1.85E-67	7.40E-28	1
0.21084805	-0.18665748	0.10197117	-0.07978234	1.05E-21	2.49E-105	5.23E-99	1
0.46903082	-0.0536463	-0.06997425	-0.09147894	3.98E-127	8.69E-194	3.52E-137	1
0.47560281	-0.07433132	0.00795974	-0.06940547	1.75E-99	1.26E-162	9.53E-128	1
