# Supplementary figures and images for: The Proneural Molecular Signature Is Enriched in Oligodendrogliomas and Predicts Improved Survival among Diffuse Gliomas
Source: PLoS One. 2010 Sep 3;5(9):e12548. doi: 10.1371/journal.pone.0012548 (PMC2933229; doi:10.1371/journal.pone.0012548)

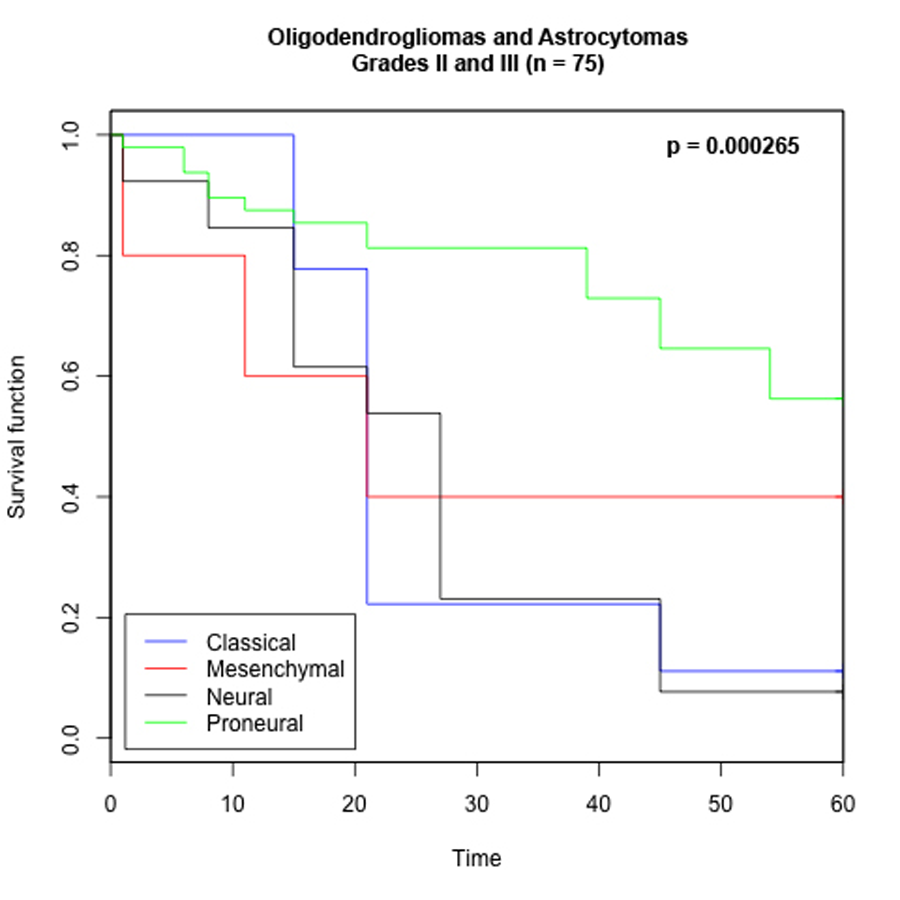

Supplement: Figure S1 — Kaplan-Meier Analysis of Low-grade gliomas. (0.13 MB TIF) [file pone.0012548.s011.tif]
